# Supplementary material for: RPIPLM: Prediction of ncRNA-protein interaction by post-training a dual-tower pretrained biological model with supervised contrastive learning
Source: PLoS One. 2025 Aug 14;20(8):e0329174. doi: 10.1371/journal.pone.0329174 (PMC12352837; doi:10.1371/journal.pone.0329174)
Supplement: Attachment — Submitted filename: Response to Reviewers.pdf [file pone.0329174.s001.pdf]

Authors' Response to Reviews of

RPIPLM: prediction of ncRNA-protein interaction by post-training a dual-tower pretrained biological model with Supervised Contrastive Learning

,

RC: ***Reviewers' Comment***, AR: Authors' Response, ☐ Manuscript Text

1. Reviewer #1

We express our gratitude to the esteemed reviewer for providing constructive feedback and valuable suggestions regarding the positive evaluation of our work. Furthermore, his/her insightful recommendations have significantly contributed to improving the quality and rigor of our manuscript.

1.1. Comment 1

RC: ***In Figure 1, the protein sequence only has A C G U. But generally there are 20 different amino acid residues. This may confuse some readers. Also the protein sequence looks identical to RNA sequence. Which may falsely make some readers think sequences can be identical. (Yes, the '...', means anything can be there but many readers may skip this). Also in the FIgure where MLM is shown the sequences do not match the input.***

AR: Thank you very much for your valuable feedback on our submitted manuscript. Due to an oversight, we mistakenly labeled an RNA sequence as a protein sequence. In the revised manuscript, we have corrected this representation error. Additionally, errors in the MLM figure have also been rectified. We appreciate your corrections and have taken the necessary steps to ensure the accuracy and clarity of the paper.

1.2. Comment 2

RC: ***In section 2.2, some concatenation methods are stated and what each concatenation method captures is given, such as , "This method provides a straightforward approach to concatenate RNA and protein features, but may not capture more complex interactions between RNA and protein." - what is the basis of the underlined states? Similarly for other concatenation methods such a statement is given. If there were references it would be better.***

AR: In our study, we explored various methods of concatenating feature vectors. Direct concatenation lacks any computation of inter-feature relationships, and is thus considered devoid of feature interaction. Conversely, other concatenation methods involve vector operations such as addition, subtraction, multiplication, and division, which are commonly referred to as feature fusion in machine learning. The outcomes of this fusion are deemed as independently fused information. Our experimental results also demonstrate that incorporating fusion information leads to improved model performance. The approach to combining feature vectors in our study is inspired by the work "Sentence-BERT: Sentence Embeddings using Siamese BERT-Networks," and in response to the reviewer's suggestion, we will include this reference in our bibliography.

1.3. Comment 3

RC: ***In section 2.3.2, kernel size of convolutional layers, filter count and depth is given which were fixed during tests. It would be better if multiple tests were done with different values for these hyperparameters.***

AR: In Section 2.3.2, we acknowledge your inquiry regarding the fixed values for the kernel size, filter count, and depth of the convolutional layers. In fact, we conducted a series of grid-cross experiments, exploring various combinations of hyperparameters. Ultimately, we selected the set of hyperparameters that demonstrated the optimal performance.

1.4. Comment 4

RC: ***In section 3, it is assumed that, if ncRNA "Rs and Ri share more than 80% sequence identity" or proteins "Ps and Pi share more than 40%" sequence identity" than if pair Rs and Ps interact then Ri and Pi interact. This way the dataset was filtered. What is the basis of choosing these percentages (80% and 40%)***

AR: We consulted the paper titled 'RPITER: A Hierarchical Deep Learning Framework for ncRNAProtein Interaction Prediction' and implemented the aforementioned data filtering approach. The primary objective of this approach is to reduce homogeneity among the data, aiming to minimize potential discrepancies between experimental results and real-world scenarios.

1.5. Comment 5

RC: ***In section 4.7, we see there were tests with No pre-training and no CL. It is unclear how such this model was trained and tested.***

AR: A non-pretrained model refers to a model trained solely on a Transformer network, utilizing contrastive learning loss in addition to cross-entropy loss as the classification objective. On the other hand, a model without contrastive learning (CL) refers to a pre-trained Transformer model that, during task-specific training, does not incorporate contrastive learning loss but relies solely on cross-entropy loss.

1.6. Comment 6

RC: ***RNABERT used RNA structural information in its pipeline. For protein encoding pipeline addition of such options can be great, as huge protein structural databases are available.***

AR: Thank you for your insightful comments. We highly appreciate your recognition of the potential value in incorporating protein structural information into our research. While we acknowledge the significance of utilizing such data, it is true that handling protein structure information often involves additional data processing and computational resources.

In our forthcoming work, we plan to address this by attempting to integrate protein structure information, introducing relevant options in the model's encoding pipeline. We recognize the crucial role this step plays in enhancing model performance and accuracy. Additionally, we intend to explore leveraging existing extensive protein structural databases to ensure the reliability and breadth of our data.

1.7. Comment 7

RC: ***There are great tools such as Hmmer or HH-Suite that can create MSA from or do profiling for protein sequences. What happens when results of these tools are used to again tune the MLM encoded results? Such tasks may improve performance.***

AR: Thank you for providing us with valuable insights. We find the idea you suggested truly exciting, and we believe it could significantly enhance our understanding of proteins. In our future work, we will certainly explore this avenue.

2. Reviewer #2

2.1. Comment 1

RC: ***In the second paragraph, some of the examples were repeated in the sentence "These interactions are involved in a range of biological processes, including disease development, cellular signaling, and gene expression regulation, among others.". Either provide new examples or remove these lines.***

AR: Thank you for your valuable feedback. We have duly noted your suggestion and have removed the repetition in the sentence: "These interactions are involved in a range of biological processes, including disease development, cellular signaling, and gene expression regulation, among others.

2.2. Comment 2

RC: ***The introduction section is over-sized. Too much focus on previous work. It is better to reduce the number of examples and their contributions. Include one/two from machine learning and two/three from deep learning with the most ground breaking contributions at their time.***

AR: Thank you for your constructive feedback. We appreciate your suggestion to streamline the introduction section. In response to your recommendation, we will reduce the number of examples and their contributions, focusing on one or two significant contributions from machine learning and two or three from deep learning, emphasizing their groundbreaking impact at their respective times.

2.3. Comment 3

RC: ***There are other model for protein feature extraction like ESM and ProteinBERT. Why have those not been tested?***

AR: Thank you for your thoughtful feedback. As you rightly pointed out, there are various protein pre-trained models available, including ESM and ProteinBERT. We appreciate your inquiry into why these models were not included in our testing.

Our decision to focus on a single pre-trained model stems from two main considerations. Firstly, our research primarily aims to present a dual-tower computational framework and validate its feasibility for studying macromolecular interactions. Within this framework, any pre-trained model can be employed. Secondly, individually testing each model entails a considerable cost, and the nuanced differences among various protein pre-trained models may not significantly impact the outcomes of our specific study.

2.4. Comment 4

RC: ***To calculate |r-p| and r*p, both r and p need to be of same size. However, the pretrained models don't give output of same size. How did the authors convert them to same size? Dense layer or 1D conv? Need to mention that.***

AR: Thank you for your insightful observation. We appreciate your attention to detail. To address the chal-

lenge of different output sizes from the pretrained models, we employed one-dimensional convolution (1D convolutions) to scale the dimensions of the features, ensuring consistency between |r-p| and r*p.

In the revised manuscript, we will explicitly mention this crucial step, highlighting the use of 1D convolutions to align the sizes of the features for accurate computation of |r-p| and r*p.

2.5. Comment 5

RC: ***The global and local attention methods were not described. From the figure it seems, global attention is scaled dot product attention by Vaswami and local attention is regular dot product attention. Should detail on these and refer to the authors of the original work.***

AR: Thank you for your feedback. We appreciate your guidance. We will follow your suggestion and provide additional references for the global and local attention mechanisms in the revised manuscript.

2.6. Comment 6

RC: ***Please highlight the best scores in Table 3.***

AR: Thank you for your suggestion. We will make the necessary revisions to Table 3 to highlight the best scores for better clarity in the revised manuscript.
