# Supplementary material for: RPIPLM: Prediction of ncRNA-protein interaction by post-training a dual-tower pretrained biological model with supervised contrastive learning
Source: PLoS One. 2025 Aug 14;20(8):e0329174. doi: 10.1371/journal.pone.0329174 (PMC12352837; doi:10.1371/journal.pone.0329174)
Supplement: Attachment — Submitted filename: Response to Reviewers.docx [file pone.0329174.s002.docx]

**Authors’ Response to Reviews of**

RPIPLM: prediction of ncRNA-protein interaction by post-training a dual-tower pretrained biological model with Super- vised Contrastive Learning

*,*

**RC: *Reviewers’ Comment***, AR: Authors’ Response, □ Manuscript Text

We express our gratitude to the esteemed reviewer for providing constructive feedback and valuable suggestions regarding the positive evaluation of our work. Furthermore, his/her insightful recommendations have significantly contributed to improving the quality and rigor of our manuscript.

**1. Reviewer #1**

**1.1. Comment 1**

**RC: *All the comments have been addressed properly. Relevant references were added and the manuscripts was properly updated. I am happy with the revised manuscript.***

AR: Thank you for your positive feedback and for your time and effort in reviewing our manuscript.

**2. Reviewer #2**

**2.1. Comment 1**

**RC: *Table-3 needs to show the best score per column in bold.***

AR: Thank you for your suggestion. We have made the necessary revisions to Table 3 to show the best score per column in bold in the revised manuscript.

**2.2. Comment 2**

**RC: *The authors need to show the applicability of the method on SOTA PLMs like ESM(https://github.com/***

***facebookresearch/esm) and RNA LMs like RiNALMo (https://arxiv.org/pdf/2403.00043) to prove robustness of the idea.***

AR: Thank you for your valuable suggestion. We agree that testing on state-of-the-art PLMs such as ESM and RiNALMo would further demonstrate the robustness and generalizability of our method. As these models have different input formats and computational demands, we plan to include them in our future work to extend RPIPLM’s applicability. In the current version, we have focused on integrating and evaluating a range of competitive PLMs (e.g., ProtBert, OntoProtein, RNABERT) to establish the foundational performance of our dual-tower framework.

**2.3. Comment 3**

**RC: *One major issue with the method is the application of dot product attention and scaled dot product attention on the concatenated features. Attention is usually applied along the length of a sequence. However, this method applies it along the feature dimension. The article never explained the rationale behind it If it wasn't the case, the description is not well written to reflect that.***

AR: We appreciate the reviewer’s insightful comment. However, we believe there may have been a misunderstanding. In our method, the dot-product attention is applied along the sequence (token) dimension, not along the feature (embedding) dimension.

As described in Section Global and Local Attention Modules (page 7), we define our input $X = [x_1, x_2, \dots, x_n]$ as a sequence of embeddings, where $n$ denotes the sequence length (i.e., the number of tokens after concatenation of RNA and protein CLS or pooled embeddings). The scaled dot-product attention then computes attention weights between all pairs of tokens in this sequence. To avoid further confusion, we have revised the manuscript to explicitly state the dimension along which attention is applied, and we have clarified that the attention is not across feature channels.

**2.4. Comment 4**

**RC: *Table-6 in Ablation studies should indicate the best scores in bold. Additionally, authors might reconsider a different title for the section. Additionally, in the description, instead of rehashing what the table shows, authors may instead focus on the improvement ( in percentage or other measures).***

AR: Thank you for your insightful observation.Thank you for the constructive suggestions regarding the ablation study section. We have updated Table 6 by highlighting the best-performing scores in bold for clearer comparison. Additionally, we have renamed the section to “Ablation Study and Performance Impact” to better reflect its content. In the revised description, we now focus on the relative performance changes, reporting the percentage improvements and degradations caused by the removal of key modules, rather than restating the table values.

**2.5. Comment 5**

**RC: *Additionally, while the difference in performance may not be big as seen in the ablation studies, it would be interesting to see the effects of the various attention modules on the leaned representations. Authors may perform the TSNE plot experiments to show that. Also, if there are significant differences, an interesting study would be exploring how the attention modules impact the feature space.***

AR: We thank the reviewer for the valuable suggestion to explore the impact of attention modules on the learned representations. While we initially planned to use t-SNE visualizations, we agree that a quantitative summary better complements our existing evaluation style.

To this end, we conducted embedding-level ablation experiments under the same concatenation scheme ($(r, p, |r - p|)$), where we removed either global attention, local attention, or both. The classification results using the derived embeddings are reported in the following table.These results show that removing either attention module consistently degrades performance, with the most notable drop occurring when both are removed. This supports the view that the attention mechanisms help shape a more discriminative and informative feature space, even when the top-line metrics are not drastically different.

| Configuration | Accuracy (NPInter2.0) | MCC (NPInter2.0) | Accuracy (RPI2241) | MCC (RPI2241) |
| --- | --- | --- | --- | --- |
| No Global Attention | 0.931 | 0.875 | 0.812 | 0.667 |
| No Local Attention | 0.922 | 0.868 | 0.801 | 0.643 |
| No Global & Local Attention | 0.903 | 0.847 | 0.785 | 0.620 |
