# Supplementary material for: RPIPLM: Prediction of ncRNA-protein interaction by post-training a dual-tower pretrained biological model with supervised contrastive learning
Source: PLoS One. 2025 Aug 14;20(8):e0329174. doi: 10.1371/journal.pone.0329174 (PMC12352837; doi:10.1371/journal.pone.0329174)
Supplement: Attachment — Submitted filename: Response_to_Reviewers_auresp_3.docx [file pone.0329174.s003.docx]

**Authors’ Response to Reviews of**

RPIPLM: prediction of ncRNA-protein interaction by post-training a dual-tower pretrained biological model with Super- vised Contrastive Learning

*,*

**RC: *Reviewers’ Comment***, AR: Authors’ Response, □ Manuscript Text

We express our gratitude to the esteemed reviewer for providing constructive feedback and valuable suggestions regarding the positive evaluation of our work. Furthermore, his/her insightful recommendations have significantly contributed to improving the quality and rigor of our manuscript.

**Reviewer and Editor**

**1. Comment 1**

**RC: *Because the model is trained in a fully supervised manner, its final embedding layer is inherently optimized for class separation, so any t-SNE projection will directly mirror its classification accuracy. As a result, the only distinction between the RPIPLM and RPITER t-SNE visualizations arises from their differing accuracy ( and other metrics) scores, and the “new” plots offered are in fact identical to those already shown in Figure 3. Additionally, the 3rd and 4th plots in the tSNE plots are reflective of the final plot in Figure 5.***

**AR:**

We thank the reviewer for the thoughtful comment and agree with the observation that “any t-SNE projection will directly mirror its classification accuracy.” Indeed, as our model is trained in a fully supervised manner, the embedding space is naturally optimized for class separation, and the resulting t-SNE plots primarily reflect this objective.

However, we would like to clarify two points:

1.We are not entirely certain about the reference to “the RPITER t-SNE visualizations”, as RPITER was not included in any t-SNE comparison in our manuscript. Furthermore, we did not introduce any new t-SNE plots in the revised version of the manuscript beyond what was already present in the original submission (Figure 6). If there has been a misunderstanding on our part, we would be grateful for clarification.

2.Regarding the comment that “the 3rd and 4th plots in the t-SNE plots are reflective of the final plot in Figure 5,” we believe this refers to a perceived overlap in visual information. We note that the last plot in Figure 5 shows the performance of RPIPLM on the RPI369 dataset, and the t-SNE example we included in Figure 6 is likewise drawn from RPI369. Therefore, the similarity is expected and intentional: the t-SNE plot serves as a geometric visualization corresponding to the same experimental condition.

**2. Comment 2**

**RC: As for what the authors can do to show the interpretability of the model They can use the attention wits to see which tokens the model is paying most attention to while classifying and if those tokens have patterns that might represent something biologically. Biologically interpret whether the model’s attention weights are indicative of the binding sites, and if the weights can shed some light on that.**

**Note that authors are not restricted to these ideas and can explore any other ideas they might find a good fit.**

**AR:**

We appreciate the reviewer’s insightful suggestion regarding the use of attention weights for biological interpretability. Indeed, analyzing which tokens receive high attention may help identify sequence regions associated with binding activity. While preliminary inspection of attention maps shows that the model occasionally highlights regions near known binding sites, a more rigorous biological validation would require curated site-level annotations, which we plan to incorporate in follow-up studies.
